# Supplementary material for: Thermo-optic epsilon-near-zero effects
Source: Nat Commun. 2024 Jan 26;15:794. doi: 10.1038/s41467-024-45054-z (PMC10817958; doi:10.1038/s41467-024-45054-z)
Supplement: Supplementary file 1 — Supplementary Information [file 41467_2024_45054_MOESM1_ESM.pdf]

Supplementary information for:
Thermo-optic epsilon-near-zero effects

Jiaye Wu[1,*], Marco Clementi[1], Chenxingyu Huang[2,3], Feng Ye[2], Hongyan Fu[3], Lei Lu[2], Shengdong Zhang[2], Qian Li[2,*], and Camille-Sophie Brès[1,*]

[1]École Polytechnique Fédérale de Lausanne (EPFL), Photonic Systems Laboratory (PHOSL), STI-IEM, Station 11, Lausanne CH-1015, Switzerland.
[2]School of Electronic and Computer Engineering, Peking University, Shenzhen 518055, China.
[3]Tsinghua Shenzhen International Graduate School, Tsinghua University, Shenzhen 518055, China.
[*]Corresponding authors. Email: jiaye.wu@epfl.ch; liqian@pkusz.edu.cn; camille.bres@epfl.ch

This file provides supplementary information for article "*Thermo-optic epsilon-near-zero effects*".

Supplementary Note 1 Ellipsometry measurements

Supplementary Note 1.1 Verification of oxygen diffusion in the grain boundary barrier model

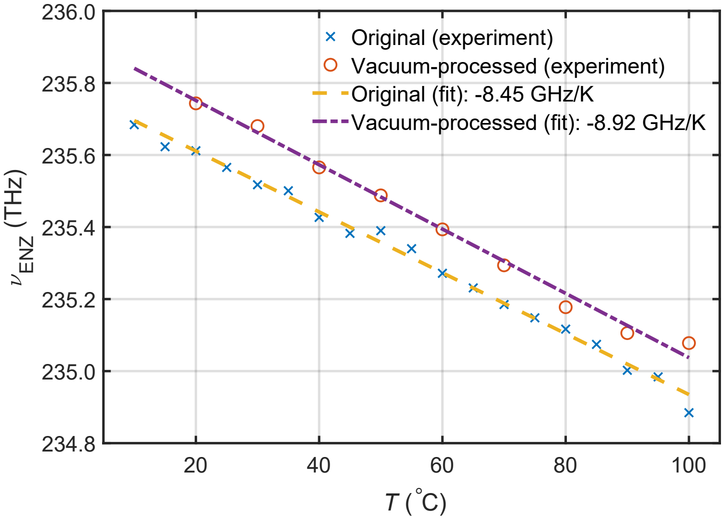

Supplementary Figure 1. Ellipsometry data comparison of O-band sample without and with vacuum pre-processing.

To verify the oxygen diffusion theory in the grain boundary barrier model, here we take the high-carrier-concentration O-band sample as an example and conduct a proof-of-concept experiment.

The exact same O-band sample is placed in a vacuum of 150 mbar for 35 minutes to lower the amount of potential oxygen inside the ITO lattice (desorption by vacuum). The sample is then measured quickly under the ellipsometer. The data in Supplementary Fig. 1 shows that the corresponding carrier concentration (in terms of ν_{ENZ}) increases, which agrees with the prediction given by a larger N_t with less oxygen occupation. Additionally, the sample shows a slightly larger temperature dependence, from -8.45 GHz·K^{-1} to -8.92 GHz·K^{-1}, owing to a larger initial N_t created by the vacuum pre-processing.

Supplementary Note 1.2 Temperature dependence of real and imaginary parts of permittivity

The experimentally measured real and imaginary parts of the permittivity near the ENZ frequencies are shown with four different temperatures in Fig. 2. For the O-band and C-band, curves with the temperatures of 25, 50, 75, 100 °C are illustrated. For the 2-μm-band, annealing happens near 80 °C, therefore, we select four other temperatures with equal distance, namely, 20, 40, 60, 80 °C. The general changes are consistent: with the increment of temperature, ε_r and ε_i increase. The ε_r variation can be understood as another aspect to observe ν_ENZ change, while the rise of ε_i does not necessarily mean an increase in total loss. The assessment of the loss should be based on the parameter α_dB, which is analyzed in detail in the main text.

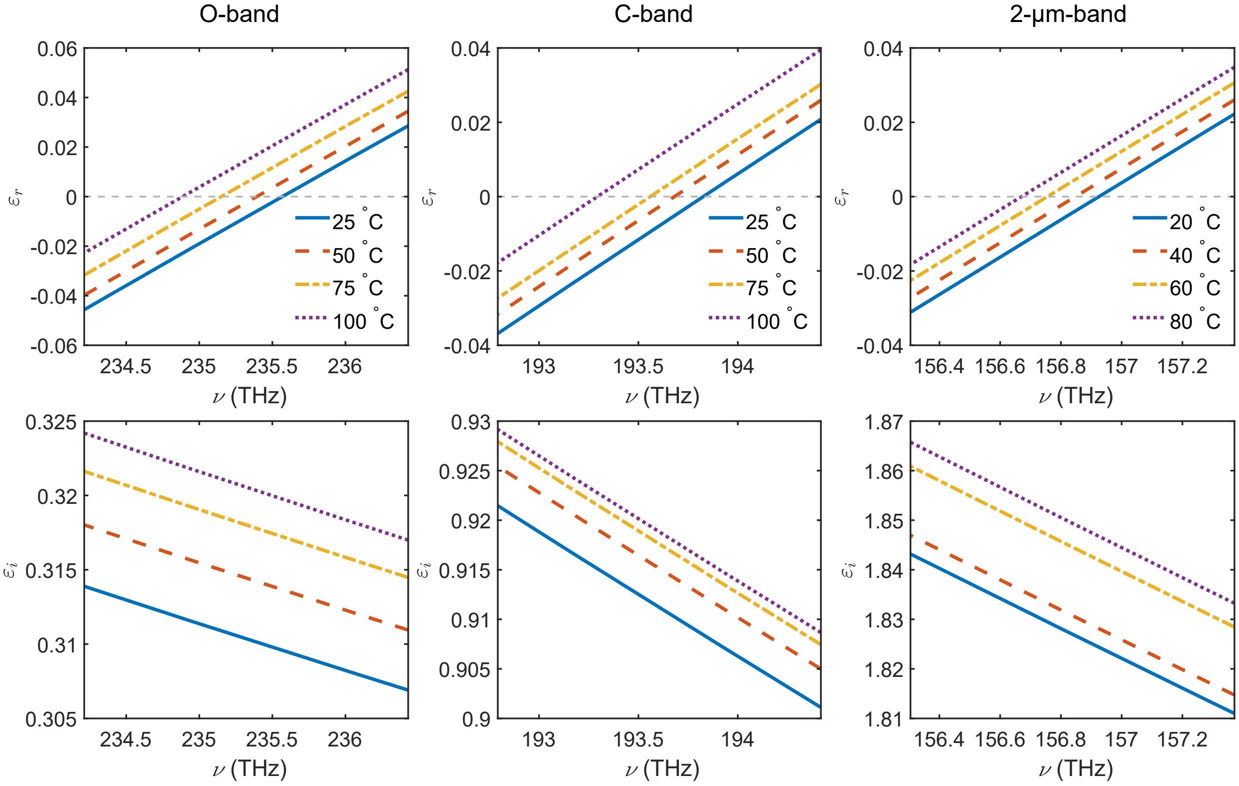

Supplementary Figure 2. The variation of real and imaginary parts of the permittivity with the temperature near the ENZ frequency of O-band, C-band, and 2-μm-band samples measured by an ellipsometer.

Supplementary Note 2 Thermal resistance network: for experiments and reference designs

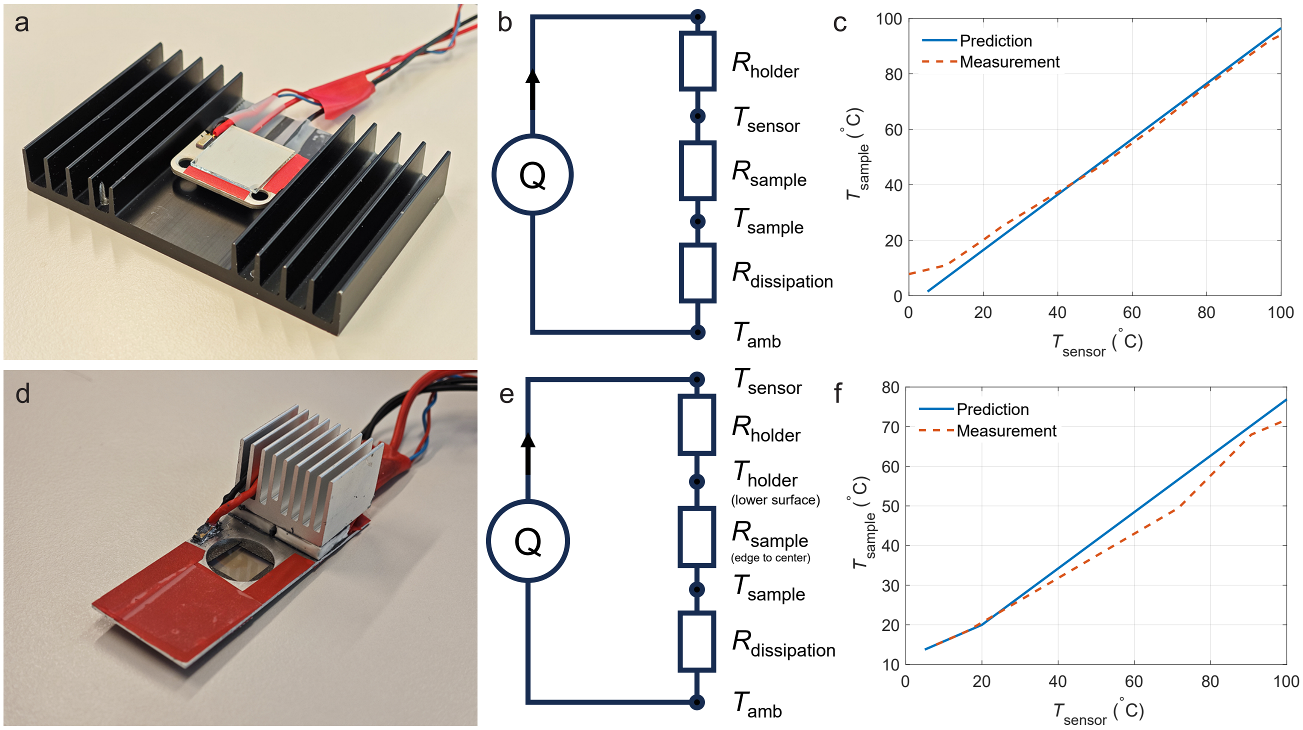

Supplementary Figure 3. Thermal designs. a A photograph of an ellipsometry-compatible sample stage; **b** Corresponding thermal resistance network of **a**; **c** Heating efficiency of **a**; **d** A photograph of a free-space-optics-compatible sample stage; **e** Corresponding thermal resistance network of **d**; **f** Heating efficiency of **d**.

One can use the "Ohm-law-like" thermal resistance network to conveniently assess heat transfer in some structures[1]. The heating power Q is analogous to the electrical current I, the thermal resistance R is analogous to the electrical resistance R, while the temperature T corresponds to the voltage U. Therefore, one has:

$$\Delta T = R_{\mathrm{concerned}} Q. \tag{S1}$$

For slabs with thickness d, the thermal resistance is:

$$R = \frac{d}{\kappa A}, \tag{S2}$$

where κ is the heat conductivity, and A is the surface area.

For circular objects like the window in Supplementary Fig. 3d, we consider a measurement area of $r_1 = 1$ mm at the center (also the size of the thermal couple), the thermal resistance is therefore,

$$R = \frac{\ln(r_2 - r_1)}{2\pi L \kappa}, \tag{S3}$$

with the r_2 being the radius of the window-opening, L being the thermal conduction thickness, *i.e.*, the thickness of the sample in this case.

For the design in Supplementary Fig. 3a, assuming a high temperature and the Peltier module is working at maximum power in Supplementary Fig. 3b,

$$T_{\text{sensor}} - T_{\text{sample}} = R_{\text{sample}} Q, \tag{S4}$$

where R_{sample} is with large A (2 cm × 2 cm) and small d (1.1 mm thickness). The heat only needs to travel through the 1.1 mm to achieve uniform heating. In this design, the heating efficiency is even not related to the material and thickness of the aluminum holder, since the T_{sensor} which is taken as the baseline of temperature difference calculation, is over the aluminum plate, and within the capability of the Peltier module and the temperature controller, T_{sensor} can be achieved as desired.

In practice, the only way to further improve this efficiency is by reducing the thickness of the sample substrate, for example, using a silicon substrate. In order to be compatible with ellipsometry, the surface of the thin film must face upwards. This is the reason why we call this model "almost universal" and suitable to serve as a design and engineering reference.

We take the following values of κ: $\kappa_{\text{Al}} = 247$ W·m^{-1}·K^{-1}, $\kappa_{\text{SiO}_2} = 4$ W·m^{-1}·K^{-1} and $\kappa_{\text{ITO}} = 1.3$ W·m^{-1}·K^{-1}. Here it is obtained that $R_{\text{sample}} = 0.688$ K·W^{-1}, yielding a temperature difference of 3.51 °C, which is consistent with the maximum measured difference of 4 °C shown in Supplementary Fig. 3c (considering AD590KF has an error of ±1 °C).

We also provide a reference design of free-space-optics-compatible sample stage with temperature control, shown in Supplementary Fig. 3d. Many free space optics experiment, such as the z-scan, saturable absorption, *etc.*, requires transmission measurement of the sample, therefore there is an opening cut to the aluminum plate.

If one calculate the thermal resistance using the aforementioned formulae, it can be found that the largest resistance exist on the sample surface, from the edge of the window-opening to the center where the experimental light beam will travel through. This is the reason why we do not put the sample on the top of the aluminum plate as Supplementary Fig. 3a does. It is better to make heat travel through 1 mm of aluminum holder than 1.1 mm of silica glass. In this case, our design reference has

$$T_{\text{sensor}} - T_{\text{sample}} = (R_{\text{holder}} + R_{\text{sample,window}}) Q, \tag{S5}$$

whose temperature difference can be 23.11 °C with $R_{\text{holder}} = 0.018$ K·W^{-1} and $R_{\text{sample,window}} = 2.25$ K·W^{-1} ($r_2 = 7.5$ mm). The measured results are consistent but not as stable as predicted (Supplementary Fig. 3f). The disadvantage of the small heat sink necessary for counter-weight and vertical holding considerations limits its heating efficiency, unlike the former design which uses the ellipsometer as a huge heat sink. Although this flaw exits, it is sufficient for our applications presented in the text.

Supplementary Note 3 Heating efficiency: temperature response time estimation

Here, we perform a rough estimation of the temperature response time. The response time is defined as the time from the readings of the temperature controller reaches $T_{\text{sensor}} = T_{\text{setting}}$ to the actual temperature at the center of the sample reaches $T_{\text{sample}} = T_{\text{sensor}}$. The heat conduction is not instantaneous, and a temperature difference (latency) remains before the target $T_{\text{sample}} = T_{\text{sensor}}$ is met. We define a very short time dt such that every component in the thermal network between the two nodes T_{sensor} and T_{sample} is elevated by dT and can be regarded as equal-temperature:

$$Q_{\text{Peltier}}\mathrm{d}t = C_{\text{R}} m_{\text{R}} \mathrm{d}T. \tag{S6}$$

where C is the heat capacitance corresponds to the resistance R, m is the mass of the resistance. We do integral on this equation over 1 °C in order to be detectable by the AD590KF sensor, and we take $C_{\text{SiO}_2} = 740$ J·kg^{-1}·K^{-1}, $m_{\text{SiO}_2} = 0.326$ g, and find that d$t = 169.2$ ms. The 2-minute temperature stabilization time given in the main text is hence sufficient.

Supplementary Note 4 Temperature dependence of carrier concentration, resistivity, mobility, and reflection

Free carrier concentration. The rate of temperature modulation on free carrier concentration N is estimated to be -7.12×10^{16} $\mathrm{cm^{-3}K^{-1}}$, -5.16×10^{16} $\mathrm{cm^{-3}K^{-1}}$, and -2.70×10^{16} $\mathrm{cm^{-3}K^{-1}}$ for the O-band, C-band, and 2-μm-band samples, respectively, which is shown in Supplementary Fig. 4. This rate decreases with lower N. The N values are extracted using the Drude model[2]. The high carrier concentration of the samples exceeds the upper limit of the Hall effect measurement system, therefore we adopt only the results from Drude extraction as a reference.

Resistivity. The resistivity of the samples can be roughly estimated by the ellipsometer model. The rate of temperature modulation on resistivity ρ is estimated to be 5.687×10^{-8} $\Omega\cdot\mathrm{cmK^{-1}}$, 1.252×10^{-7} $\Omega\cdot\mathrm{cmK^{-1}}$, and 1.242×10^{-7} $\Omega\cdot\mathrm{cmK^{-1}}$ for the O-band, C-band, and 2-μm-band samples, respectively, which is shown in Supplementary Fig. 5a. To verify this datum, we performed Hall resistivity measurements on nominally identical samples from the same fabrication batch. The results, shown in Supplementary Fig. 5b, provide a qualitatively similar trend, displaying increase, recovery, and post-annealing behaviors analogous to the ones assessed trough ellipsometry. The rate of temperature modulation on resistivity ρ is estimated with this technique to be 2.421×10^{-7} $\Omega\cdot\mathrm{cmK^{-1}}$, 1.924×10^{-7} $\Omega\cdot\mathrm{cmK^{-1}}$, and 3.288×10^{-7} $\Omega\cdot\mathrm{cmK^{-1}}$ for the O-band, C-band, and 2-μm-band samples, respectively. We note that the discrepancy between the two sets of data should be attributed to the different measurement techniques, as well as the use of different samples. In this framework, the Hall measurement should be regarded as more reliable, as it provides a direct assessment of the electrical properties, while the ellipsometric one, albeit consistent within one order of magnitude, relies on extrinsic parameters such as layer thickness and models used for the fit.

Carrier mobility. As a by-product of the Hall effect measurement, here we provide the temperature dependence of the carrier mobility of the ENZ ITO samples. The rate of temperature modulation on mobility $\mathrm{d}\mu/\mathrm{d}T$ is -0.0228 $\mathrm{cm^2V^{-1}s^{-1}K^{-1}}$, -0.009412 $\mathrm{cm^2V^{-1}s^{-1}K^{-1}}$, and -0.005202 $\mathrm{cm^2V^{-1}s^{-1}K^{-1}}$ for the O-band, C-band, and 2-μm-band samples, respectively, which is shown in Supplementary Fig. 6.

Reflectivity at normal incidence. The full spectrum of temperature dependence of reflectivity calculated from the ellipsometry data is presented in Supplementary Fig. 7. The transitions from high reflection to low reflection (high transmission) with increasing frequency. The ENZ frequency marks the transition boundary. A higher ENZ frequency gives a significantly higher reflectivity at lower frequencies.

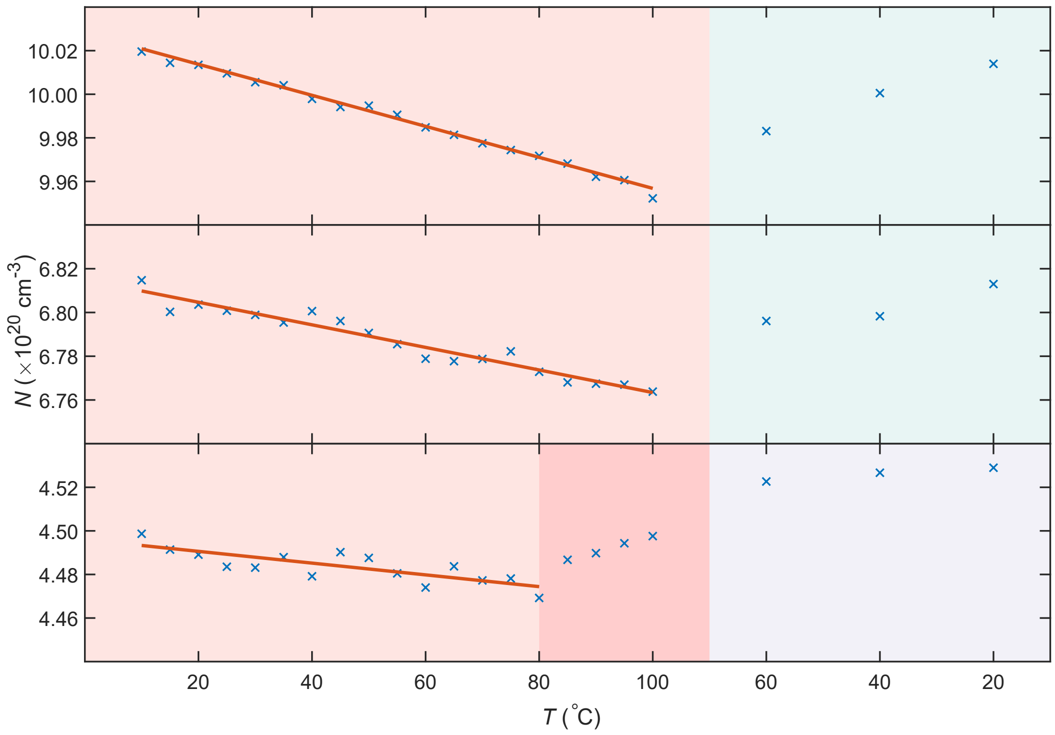

Supplementary Figure 4. Free carrier concentration comparison. The variation of free carrier concentration with the temperature at different thermal phases of O-band (top), C-band (middle), and 2-μm-band (bottom) samples.

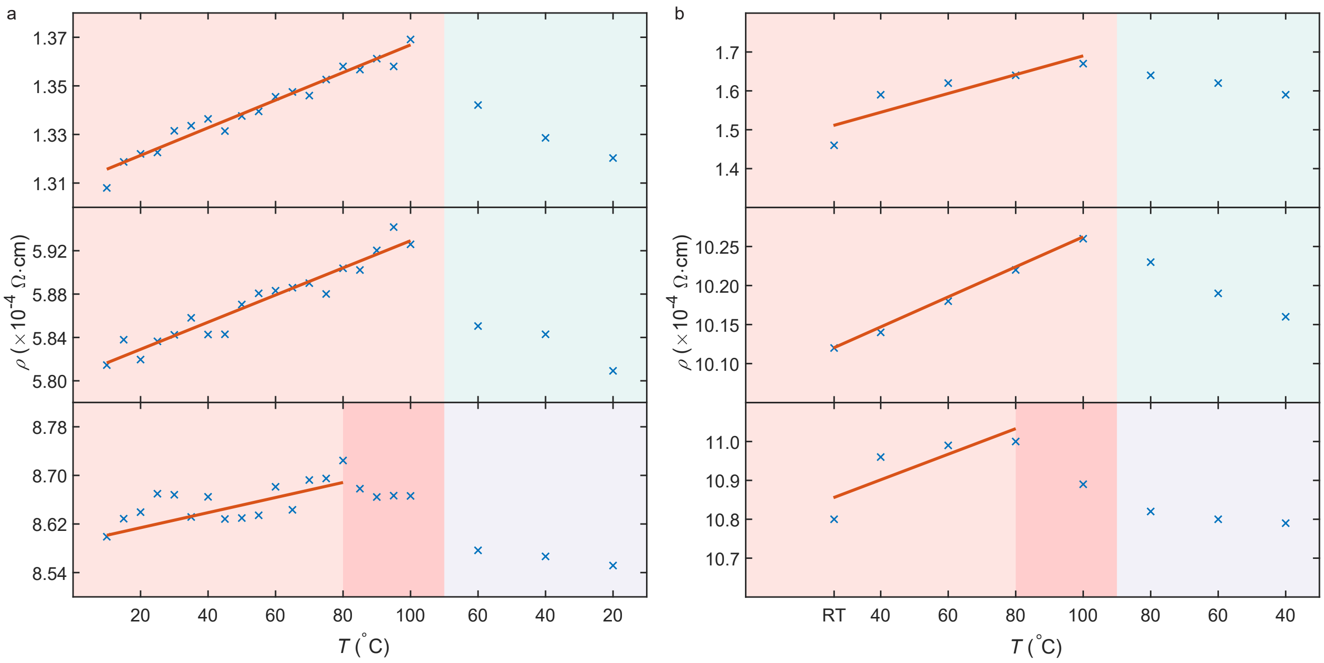

Supplementary Figure 5. Resistivity comparison. The variation of resistivity with the temperature at different thermal phases of O-band (top), C-band (middle), and 2-μm-band (bottom) samples.

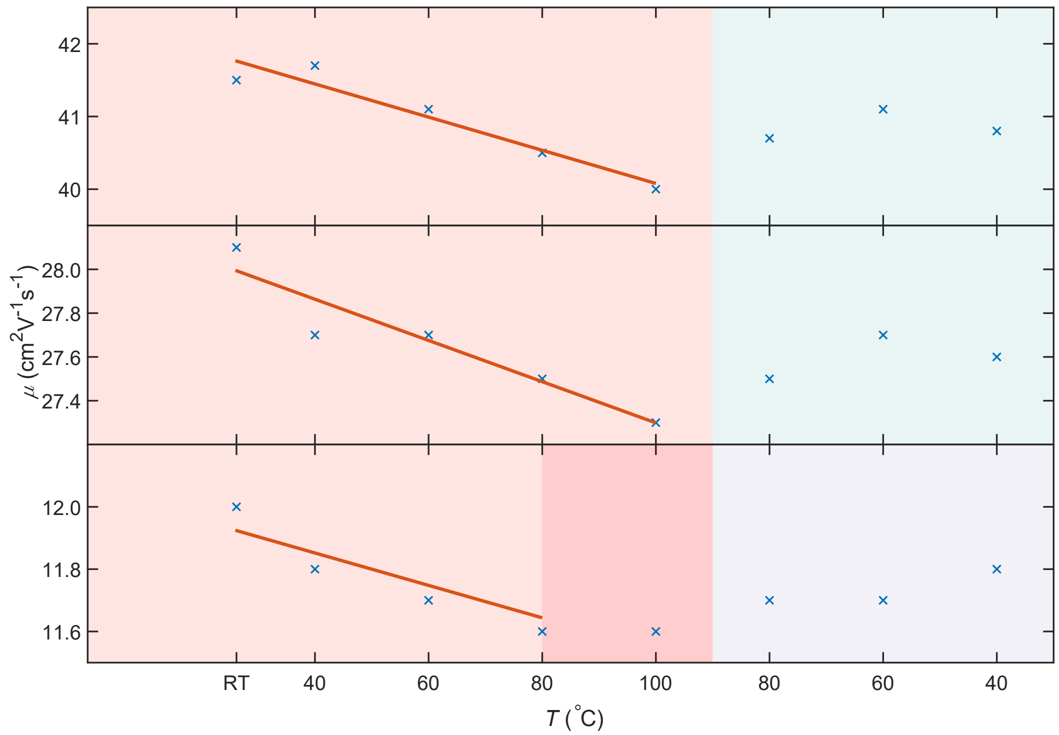

Supplementary Figure 6. Carrier mobility. The variation of carrier mobility with the temperature at different thermal phases of O-band (top), C-band (middle), and 2-μm-band (bottom) samples.

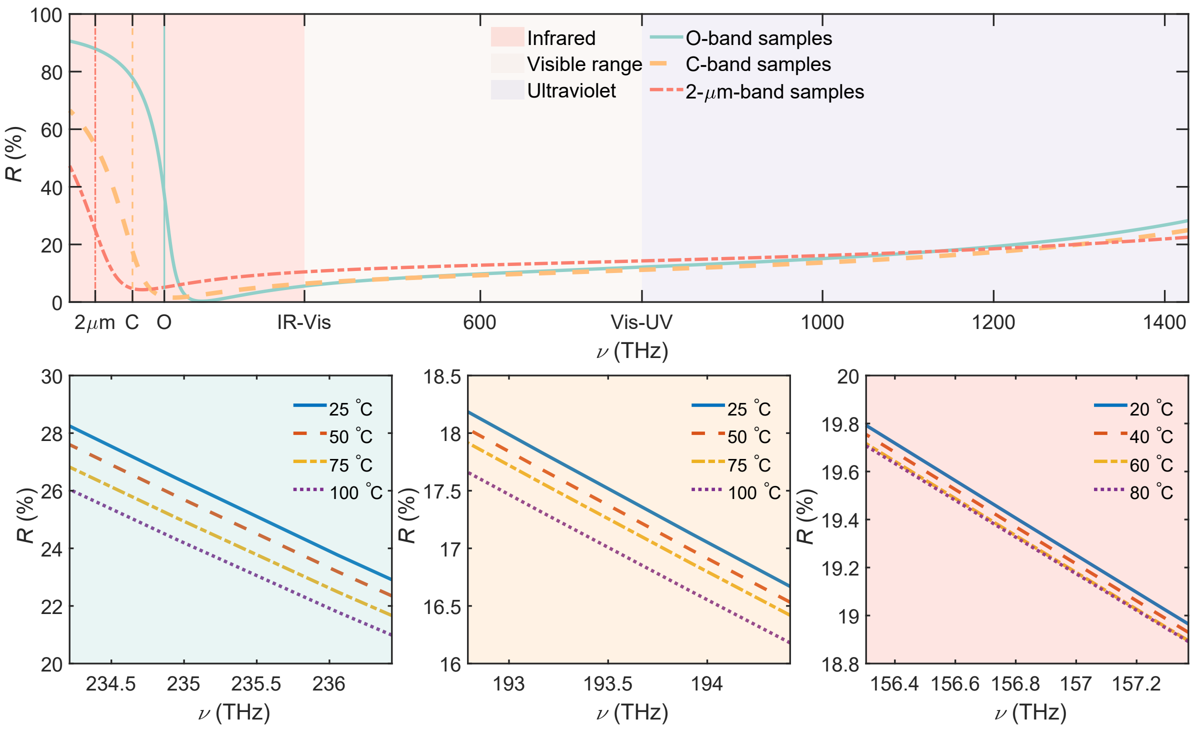

Supplementary Figure 7. Reflectivity. Full reflection spectrum of the three sets of samples. (Subfigures from left to right) Enlarged spectra in the ENZ zone of the O-band, C-band, and 2-μm-band samples. The colored vertical lines indicate the exact ENZ frequencies of the corresponding curves. On the axis of the full spectrum, Vis stands for visible light, IR is infrared, and UV is ultraviolet.

Supplementary Note 5 Estimation of the linear influence on the nonlinear Kerr effect

In ENZ TCOs, the nonlinear-index coefficient n_2 can be considered loss-driven[3–5] and carrier-dependent[6]. The relation between n_2 and the third-order susceptibility $\chi^{(3)}$ reads $n_2 = 3/(4\varepsilon_0 c n^2)\Re(\chi^{(3)})$ where $\Re$ denotes the real part operation. This is the reason why high nonlinearities with 170% n change can be located near ν_{ENZ} where loss is high and n is low[7]. Due to the relatively low-efficient nature of the free-space sample stage, light source limitations, and the lack of a wide scanning range of z-scan, a temperature-dependent $\chi^{(3)}$ measurement within the samples' respective ENZ regions can be difficult. However, we could still provide an estimation on the linear-effect-induced influence on n_2, including the combined effects of T, n (with ENZ conditions), α_{dB}, and the TOC enhancement. We take the data at room temperature 20 °C of each type as the baseline reference and we calculate the "Linear-effect-induced n_2 enhancement factor" by:

$$\frac{n_2^{(\mathrm{HT})}}{n_2^{(\mathrm{RT})}} = \frac{n_{\mathrm{RT}}^2}{n_{\mathrm{HT}}^2} \cdot \frac{\Re(\chi^{(3)})_{\mathrm{HT}}}{\Re(\chi^{(3)})_{\mathrm{RT}}} = \mathscr{H}_{\mathrm{linear}} \cdot \mathscr{H}_{\mathrm{nonlinear}}, \tag{S7}$$

where "RT" denotes room temperature and "HT" denotes the higher temperature. $\mathscr{H}_{\mathrm{linear}} = n_{\mathrm{RT}}^2/n_{\mathrm{HT}}^2$ is the considered "Linear-effect-induced n_2 enhancement factor" and $\mathscr{H}_{\mathrm{nonlinear}} = \Re(\chi^{(3)})_{\mathrm{HT}}/\Re(\chi^{(3)})_{\mathrm{RT}}$ is the temperature-dependent third-order susceptibility change.

According to the theoretical model relating loss and nonlinearity[4] in ENZ materials, the effective third-order susceptibility displays $\Re(\chi^{(3)})_{\mathrm{eff}} \propto \varepsilon_{\mathrm{b}}\alpha n\tau/(2\eta_0 N E_{\mathrm{F}})$, where α is the enhanced absorption coefficient, τ is the time of energy accumulation in hot carriers by the pulses, η_0 is the free space impedance, and E_{F} is the Fermi level. The parameters inside this expression have different tendencies when the temperature is increased, for example, due to temperature-dependent anharmonicity, thermal expansion of lattice, and the modification of band structure[8]. Therefore, we focus on the more-quantifiable linear influences, the $\mathscr{H}_{\mathrm{linear}}$ which is plotted in Supplementary Fig. 8.

In Supplementary Fig. 8, a $< 1\%$ n_2 enhancement can be found in the UV regime when heating probably due to an enhanced Lorentz resonance. While in the ENZ regime, the O-band sample experiences a 10% reduction on the linear factor, with the C-band and 2-μm-band samples showing a 3% and 2% decline, respectively. Conclusions can be drawn that higher N samples might experience a greater linear-factor-induced nonlinear performance drop at higher temperatures in the proximity of ν_{ENZ}.

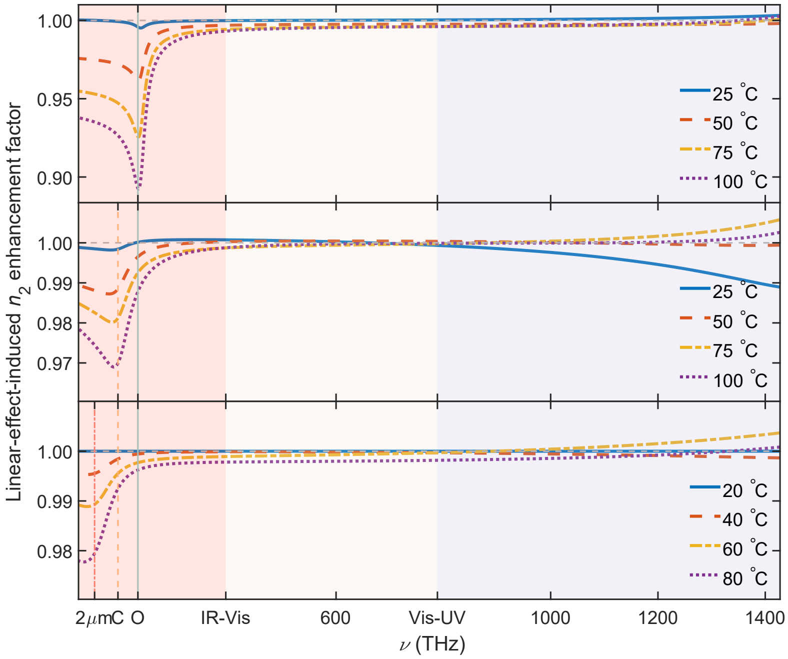

Supplementary Figure 8. Linear-effect-induced nonlinear-index coefficient enhancement factor. The three panels show the spectra of the enhancement factor of the O-band (top), C-band (middle), and 2-μm-band (bottom) samples. The colored vertical lines indicate the exact ENZ frequencies of the corresponding curves.

Supplementary Note 6 Sample information

Supplementary Note 6.1 Fabrication details

Fabrication method: 99.99% purity 10 wt% ITO target by DC magnetron sputtering.

Nominal thickness: 130 nm (actual thickness might vary with fabrication machines, techniques, precision, and annealing).

ITO samples[1]	Actual thickness[2]	Fabrication environment	Sputtering power	Annealing conditions
O-band	138.06±0.236 nm	continuous flow[3]	160 kW, 20% power	400 °C, 30 minutes in vacuum
C-band	162.91±0.666 nm	research cleanroom	150 W	350 °C, 2 hours in vacuum
2-μm-band	127.63±0.178 nm	continuous flow	160 kW, 20% power	no annealing
non-ENZ	146.49±0.116 nm	research cleanroom	150 W	no annealing

Note:

1. The electrical properties and their temperature dependence can be referred to in Supplementary Note 4.
2. Thickness obtained from ellipsometry. The deviations in thickness do not affect the results presented in this work, since both the TOC and the thermo-optic nonlinearity are thickness-independent.
3. This technique is commercial and allows for large-area deposition and large-scale high-stability production.

Supplementary Note 6.2 Thin film characteristics at room temperature: transmittance, reflectance, and absorbance

The transmittance, reflectance, and absorbance at normal incidence under room temperature for the O-band, C-band, 2-μm-band ENZ, and non-ENZ ITO samples are illustrated in Supplementary Fig. 9. The curves are calculated from the ellipsometry data and they agree with the theoretical predictions in our previous work[9].

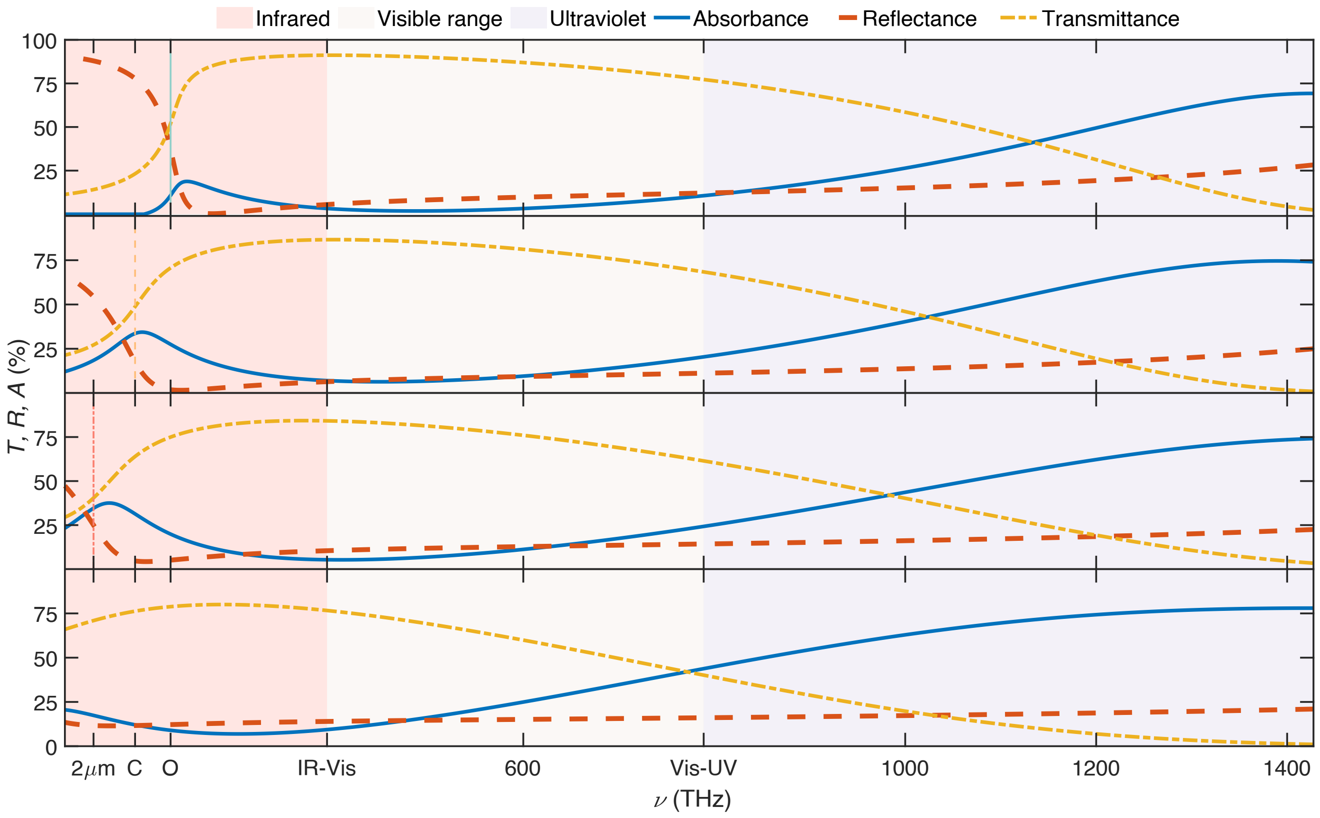

Supplementary Figure 9. The full spectra of transmittance, reflectance, and absorbance of the O-band, C-band, 2-μm-band ENZ, and non-ENZ ITO samples.

Supplementary Note 6.3 Drude parameters extraction

By the Drude model[2] described in Eq. (1), and assuming the effective mass of the electron[10] is $m^* = 0.38m_0$, the following Drude parameters can be extracted by using both the ellipsometry and the Hall effect data.

ITO samples	ε_b (dimensionless)	N (cm^{-3})	ω_{ENZ} ($rad \cdot s^{-1}$)	ω_p ($rad \cdot s^{-1}$)	γ ($rad \cdot s^{-1}$)
O-band	3.8085	1.00×10^{21}	1.48×10^{15}	2.90×10^{15}	1.12×10^{14}
C-band	3.7837	6.82×10^{20}	1.22×10^{15}	2.39×10^{15}	1.65×10^{14}
2-μm-band	3.3599	4.50×10^{20}	9.87×10^{14}	1.94×10^{15}	3.86×10^{14}

Supplementary Note 6.4 Cross-sectional scanning electron microscope (SEM) images

By the state-of-the-art SEM imaging technology, we took the cross-sectional photographs of our samples shown in Supplementary Fig. 10 as a verification of the ellipsometry results. The thickness values from the two instruments are very close (*c.f.*, Supplementary Note 6.1), indicating a sufficiently good fit of the ellipsometer model. The images are filmed by a HITACHI SU8010 cold-field emission SEM with $\times$100k magnification.

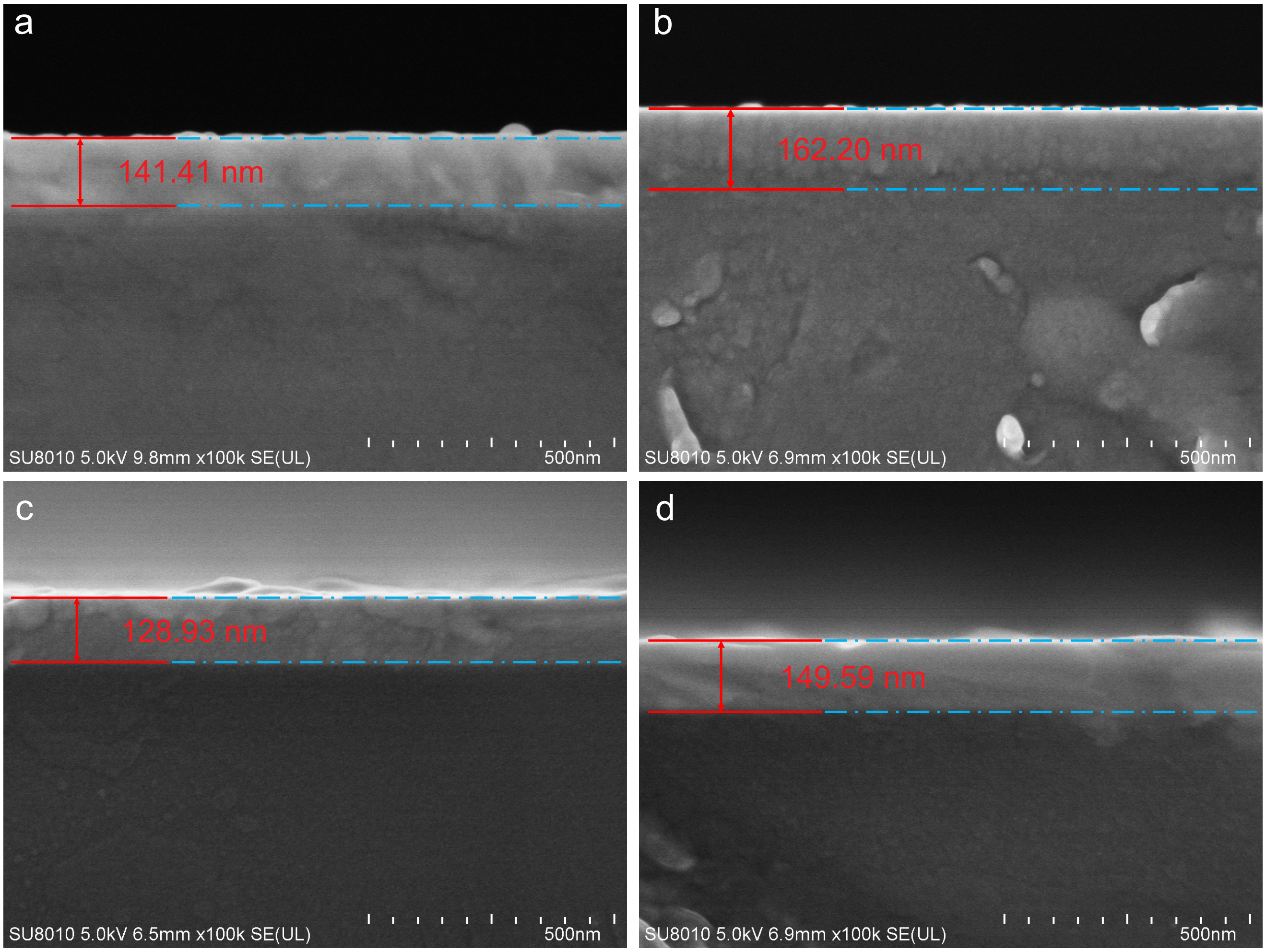

Supplementary Figure 10. The cross-sectional SEM images of the ITO samples. **a** O-band, **b** C-band, **c** 2-μm-band ENZ, and **d** non-ENZ.

Supplementary Note 6.5 Stability over additional heat-up and cool-down cycles

To test the thermal stability and degradation of the ENZ ITO samples, and to demonstrate the reproducibility of the presented experiments, we perform two extra cycles of the heat-up and cool-down processes in addition to our original data. The results are illustrated in Supplementary Fig. 11, with the O-band and C-band samples exhibiting recoverability, while the 2-μm-band samples show irreversible degradation over cycles due to the occurrence of annealing.

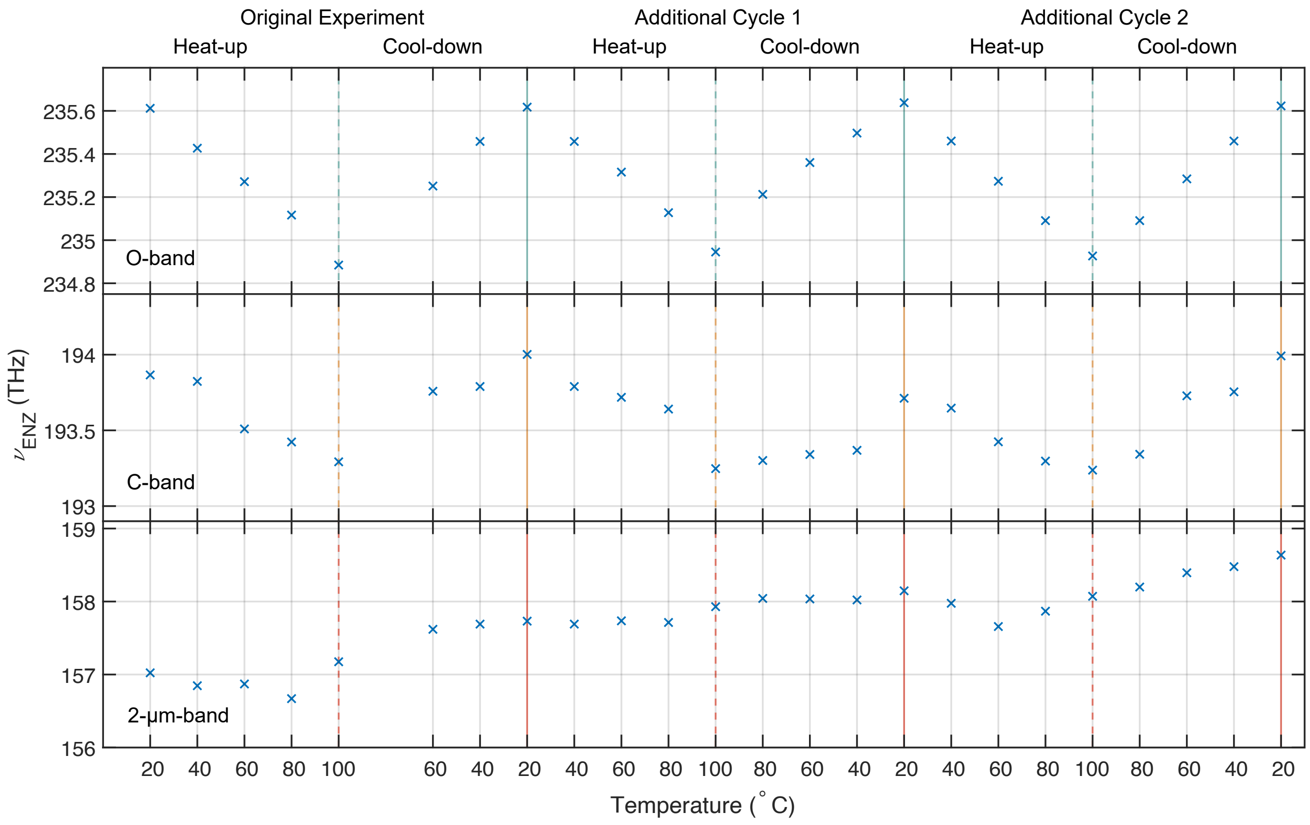

Supplementary Figure 11. The change of ENZ frequencies over additional heat-up and cool-down cycles of the O-band, C-band, and 2-μm-band ENZ ITO samples.

Supplementary Note 6.6 Uniformity assessment of samples

The fitting models of ellipsometry allow for the assessment of sample uniformity in terms of surface roughness and inhomogeneity. The non-uniformity of the samples has been taken into account in the experiments and it also provides additional information for the comparison among different fabrication methods.

ITO samples	Surface roughness	% Inhomogeneity (graded index model)	Sample type
O-band	6.16±0.131 nm	0.10	commercial
C-band	3.23±0.384 nm	11.60	research cleanroom
2-μm-band	8.12±0.116 nm	-2.05	commercial

Supplementary References

1. Çengel, Y. A. & Ghajar, A. J. *Heat and Mass Transfer: Fundamentals & Applications* (McGraw-Hill Education, New York, United States, 2015), 5 edn.
2. Drude, P. Zur elektronentheorie der metalle. *Annalen der Physik* **306**, 566–613 (1900).
3. Khurgin, J. B., Clerici, M. & Kinsey, N. Fast and slow nonlinearities in epsilon-near-zero materials. *Laser & Photonics Reviews* **15**, 2000291 (2021).
4. Secondo, R., Khurgin, J. & Kinsey, N. Absorptive loss and band non-parabolicity as a physical origin of large nonlinearity in epsilon-near-zero materials. *Optical Materials Express* **10**, 1545 (2020).
5. Minerbi, E., Sideris, S., Khurgin, J. B. & Ellenbogen, T. The role of epsilon near zero and hot electrons in enhanced dynamic thz emission from nonlinear metasurfaces. *Nano Letters* **22**, 6194–6199 (2022).
6. Elim, H., Ji, W. & Zhu, F. Carrier concentration dependence of optical kerr nonlinearity in indium tin oxide films. *Applied Physics B* **82**, 439–442 (2006).
7. Alam, M. Z., De Leon, I. & Boyd, R. W. Large optical nonlinearity of indium tin oxide in its epsilon-near-zero region. *Science* **352**, 795–797 (2016).
8. Agrawal, G. P. *Nonlinear Fiber Optics* (Academic, 2013), 5 edn.
9. Wu, J., Malomed, B. A., Fu, H. Y. & Li, Q. Self-interaction of ultrashort pulses in an epsilon-near-zero nonlinear material at the telecom wavelength. *Optics Express* **27**, 37298–37306 (2019).
10. Wu, J., Xie, Z. T., Sha, Y., Fu, H. Y. *et al.* Epsilon-near-zero photonics: infinite potentials. *Photonics Research* **9**, 1616 (2021).
